# Severe reversible cardiac failure after bortezomib treatment combined with chemotherapy in a non-small cell lung cancer patient: a case report

**DOI:** 10.1186/1471-2407-6-129

**Published:** 2006-05-11

**Authors:** Jens Voortman, Giuseppe Giaccone

**Affiliations:** 1 Department of Medical Oncology, VU University Medical Center, Amsterdam, The Netherlands

## Abstract

**Background:**

Bortezomib (Velcade^®^), a dipeptide boronate proteasome inhibitor, is a novel anti-cancer agent registered for multiple myeloma (MM). It has also shown promising clinical activity in non-small cell lung cancer (NSCLC). Clinical experience with bortezomib so far indicates that overall incidence of cardiac failure associated with bortezomib therapy remains incidental. Nevertheless, acute development or exacerbation of congestive cardiac failure has been associated with bortezomib treatment.

**Case presentation:**

We present here a case of severe, but reversible, congestive cardiac failure in a lung cancer patient who had no prior cardiac history, after receiving an experimental treatment of bortezomib combined with chemotherapy. Elevated levels of N-terminal pro-B-type natriuretic peptide (NT-proBNP), as retrospectively measured in archived serum samples, were suggestive of pre-existent (sub-clinical) left ventricular dysfunction.

**Conclusion:**

Based on literature, we hypothesize that baseline presence of sub clinical cardiomyopathy, characterized by a dysregulation of the ubiquitin-proteasome system, could have predisposed this patient for a cardiac side effect induced by systemic proteasome inhibition.

Patients with heart disease or risk factors for it should be closely monitored when being submitted to treatment with proteasome inhibition therapy such as bortezomib. Caution is therefore warranted in lung cancer patients who often present with cardiac comorbidities.

## Background

The proteasome is a large protein complex with ATP-dependent proteolytic activity. In the evolutionarily highly conserved ubiquitin-proteasome pathway (UPP), a protein is "tagged" for degradation by the proteasome, when it is labeled with a chain of multiple ubiquitin molecules [1]. Bortezomib is a reversible inhibitor of the proteasome. Inhibition results in the accumulation of poly-ubiquitinated proteins involved in a plethora of signaling pathways. Among other effects, this can trigger apoptosis, with a relative selectivity in cytotoxicity for malignant as opposed to normal cells [2].

Bortezomib, currently registered for multiple myeloma, showed some clinical activity as monotherapy in unselected non-small cell lung cancer (NSCLC) patients [3,4]. However, there is a strong preclinical rationale to investigate combination treatments of bortezomib and chemotherapy in solid tumors, including NSCLC. The patient we report here was included in a dose-finding study of bortezomib combined with cisplatin-gemcitabine chemotherapy in first-line treatment of patients with advanced solid tumors [5].

## Case presentation

A 53-year-old male patient with stage IIIB squamous cell carcinoma of the lung, not invading the great vessels or heart structures, was treated with bortezomib combined with cisplatin-gemcitabine. The patient completed four 3-week cycles of bortezomib 1.0 mg/m^2 ^on days 1 and 8, cisplatin 70 mg/m^2 ^on day 1, gemcitabine 1000 mg/m^2 ^on days 1 and 8. An impressive radiological partial response (PR) was observed after 2 cycles of therapy, and confirmed after 4 cycles, according to the Response Evaluation Criteria for Solid Tumors (RECIST) criteria [6]. The longest diameter of the primary tumor in the left lower lobe had decreased from 85 to 35 millimeters (minus 59%).

However, from cycle 2 onwards, the patient had complained of increasing fatigue, orthopnea, and dyspnea on exertion. This was most evident during the first days of each cycle, following bortezomib, gemcitabine and cisplatin administration, including cisplatin-hydration (6 liter of fluids in 27 hours). Though he had been a heavy smoker (36 pack years) and had a history of COPD he did not have a documented or symptomatic prior cardiac history.

Because of the good anti-tumor response, treatment was continued. Upon admission for start of cycle 5, auscultation of the heart had revealed no abnormalities. Pulmonary auscultation was unchanged compared to prior examinations with a diffuse prolonged expiration and occasional rales but no crepitations. Central venous pressure as determined by neck vein distension was not elevated and there was no peripheral edema. During the night he received a transfusion of two packed cells for anemia (Hb 5.4 mmol/l).

Upon completion of cisplatin prehydration (1 liter of fluids), the patient became progressively dyspnoic. He did not experience any chest pain. An ECG did not show signs of ischemia. Chest-x-ray showed an increased heart-chest ratio of 0.57 compared to baseline 0.50, and mild signs of redistribution. His dyspnea improved remarkably upon administration of furosemide.

All chemotherapy was discontinued and he was released the next day in a clinically stable condition, though fatigue and dyspnea upon exertion had considerably worsened compared to baseline. An echocardiography performed the following week showed dilatation of the left ventricle and atrium. Ejection fraction (EF) was estimated to be 10–15%. A cardiac 99mTc-MIBI scan performed two weeks later showed diffuse hypokinesis and fixed perfusion defects in the apex and inferior wall. Treatment was initiated with atenolol, lisinopril and acenocoumarol. The first three months of follow-up did not show clinical improvement, and an echocardiography performed at that time estimated EF still at 20–30%. For these reasons no consolidation radiotherapy to the primary tumor was given. Fortunately, at six months of follow-up his condition had improved to New York Heart Association (NYHA) class I-II. At that time coronary angiography was performed which did not show significant abnormalities aside from calcification of the left anterior descending artery. At eight months of follow-up, a Multiple Gate Acquisition (MUGA)-scan showed a restoration of the EF to 45%. Unfortunately, at this time progression of his lung tumor was evident and second-line therapy was started shortly after.

**Figure 1:**
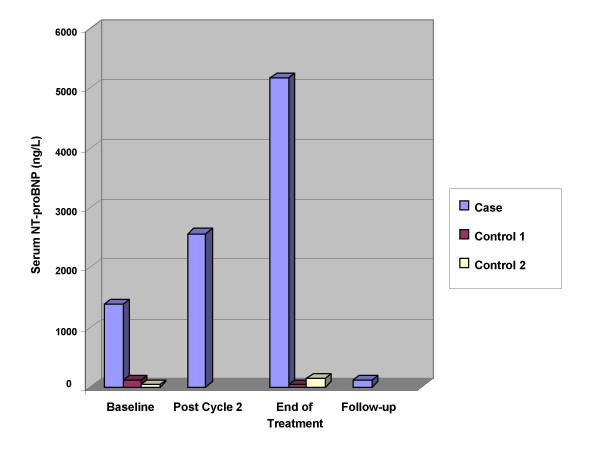
Serum levels of NT-proBNP in case report patient," case", and two control patients "control 1"and "control 2". NT-proBNP was measured in case patient at baseline (1389 ng/L), post cycle 2 (2569 ng/L), end-of-treatment (5177 ng/L) and at follow-up (110 ng/L), 10 months after end of treatment. In control patients NT-proBNP was measured at baseline and end-of-treatment only. Baseline NT-proBNP values for control patients 1 and 2 were 117 ng/L and 31 ng/L, end-of-treatment values 31 ng/L and 142 ng/ml respectively. Institutional upper limit of normal is 179 ng/L.

As we did not have a pre-treatment EF value of this patient, N-terminal pro-B-type natriuretic peptide (NT-proBNP) was determined in a pre-treatment serum sample as a surrogate marker for baseline left ventricular function (LVF). Figure 1 shows that already at baseline NT-proBNP was considerably elevated at 1389 ng/L, corresponding to a level generally found in NYHA Class II-III patients [7]. Analyzing two additional serum samples showed an increase to 2569 ng/L post cycle 2 (six weeks on treatment) and to 5177 ng/L at end-of-treatment (post cycle 4, twelve weeks on treatment) indicating a progressive worsening of the LVF during treatment. Troponin as well as CK-MB levels were not elevated in any of the samples tested. As a control, NT-proBNP at baseline and end-of-treatment was determined in two other patients participating in the same clinical trial and who had similar exposure to bortezomib and chemotherapy. NT-proBNP remained within normal limits in these two patients. Also LVEF was determined by MUGA-scan at baseline and at end-of-treatment in these two patients and found not to have decreased during treatment. A baseline and end-of-treatment MUGA-scan had been incorporated into the trial design after occurrence of the case as described above. In the case patient, at eleven months of follow-up and cardiac medication, NT-proBNP had decreased to a level within normal limits (110 ng/L).

We attribute the severe reduction of the LVEF in this case primarily to the drug treatment with bortezomib, gemcitabine and cisplatin. Main arguments are its reversibility and the negative findings of coronary angiography and additional investigations. The straining cisplatin-hydration was probably also an attributing factor, though unlikely to be the primary cause, as one would generally not expect such a slow recovery after a mere overload induced cardiac failure.

Cisplatin and gemcitabine have not been clearly associated with cardiac failure, though cisplatin has been occasionally associated with acute as well as late arterial occlusive events such as myocardial infarction and cerebrovascular thrombotic events. Cisplatin-induced alteration of platelet aggregation as well as vascular damage are possibly involved [8-10]. A study evaluating LVEF of 31 NSCLC patients immediately before and 12 weeks after finishing first-line cisplatin-gemcitabine chemotherapy did not demonstrate a significant decrease in LVEF [11].

Clinical experience with bortezomib so far indicates that the incidence of cardiac failure or other cardiovascular effects remains incidental. Nevertheless, the US package insert states that acute development or exacerbation of congestive heart failure, and/or new onset of decreased left ventricular ejection fraction has been reported [12]. It must be noted that the package insert covers adverse events observed with bortezomib monotherapy only, which does not require pre- and/or post-hydration and represents a minimal fluid load. The largest study conducted so far with bortezomib was the APEX study, a 669 patient phase 3 study, comparing bortezomib with high-dose dexamethasone in heavily pretreated patients with relapsed multiple myeloma. This study did not show a negative impact of bortezomib on the incidence of congestive heart failure, which was comparable in both arms at 2 percent [13].

There is evidence from literature that activation of the ubiquitin-proteasome system (UPS) occurs in chronic conditions of left ventricular hypertrophy and (dilated) cardiomyopathy. Increased expression of genes coding for ubiquitin-proteasome pathway was demonstrated in a rat cardiac hypertrophy model [14]. Furthermore, in dilated cardiomyopathy (DCM) patients, increased expression of regulatory proteins of the UPS was demonstrated by functional proteomics in heart tissue specimens. Activation of the UPS, leading to an altered protein expression in cardiac muscle cells, has been proposed to be an adaptive mechanism for maintaining a normal stroke volume [15]. Interestingly, susceptibility for cardiovascular disease has been ascribed to age-related decrease in proteasome activity, impairing the the ability of myocytes to mount an appropriate response to stress [16].

In this patient, baseline left ventricular dysfunction was shown by retrospective analysis of serum NT-proBNP, a peptide that is secreted by cardiac myocytes upon ventricular distension and overload. Elevated levels are related to (acute) congestive heart failure and increased long-term mortality [17]. NT-proBNP has been shown to remain stable in serum samples stored over a prolonged period of time, which allowed retrospectively analysis in remaining serum samples [18].

## Conclusion

We hypothesize that baseline presence of sub clinical cardiomyopathy, characterized by an adaptive, cytoprotective activation of the UPS, could have predisposed this patient for a cardiac side effect induced by systemic proteasome inhibition.

We recommended that patients with heart disease or risk factors for it should be closely monitored when treated with systemic proteasome inhibition therapy. Caution is therefore warranted in lung cancer patients who often present with cardiac comorbidities. Episodic measurements of NT-proBNP may be of use in this regard, however this needs to be validated in further studies.

## Competing interests

G. Giaccone would like to declare having received a speaker's fee from Millennium Pharmaceuticals, the manufacturer of bortezomib (Velcade^®^). J. Voortman declares to have no competing interests.

## Authors' contributions

JV prepared the manuscript, reviewed the literature, prepared the figure and edited the report. GG provided expert guidance throughout preparation of the manuscript and reviewed the report. GG is principal investigator of the clinical study in which the patient participated. JV and GG both participated in patient care. JV was involved in obtaining serum samples from the patient. Both authors have read and approved the final manuscript.

## Pre-publication history

The pre-publication history for this paper can be accessed here:
